# Stakeholder Analysis and Social Network Analysis in the Decision-Making of Industrial Land Redevelopment in China: The Case of Shanghai

**DOI:** 10.3390/ijerph17249206

**Published:** 2020-12-09

**Authors:** Wendong Wu, Fang He, Taozhi Zhuang, Yuan Yi

**Affiliations:** 1 School of Economics and Management, Tongji University, Shanghai 200092, China; wuwendong1007@163.com (W.W.); heyoufang@tongji.edu.cn (F.H.); yiyuan_ella@163.com (Y.Y.); 2 School of Management Science and Real Estate, Chongqing University, Chongqing 400045, China

**Keywords:** industrial land redevelopment, people’s wellbeing, decision making, stakeholder analysis, social network analysis, China

## Abstract

Currently, many large Chinese cities have entered the postindustrial era, leaving a large amount of vacant, inefficiently utilized industrial land and buildings in the inner cities. Industrial land redevelopment (ILR) can benefit cities in multiple ways, such as by increasing urban public space, improving the quality of life of citizens, and improving the environment, and is considered an effective approach to enhance people’s wellbeing. However, large-scale ILR projects often raise a series of social issues in practice, such as injustice and inequality. To address complex urban issues, ILR requires multifaceted, coordinated, and comprehensive strategies involving multitudinous stakeholders. A profound understanding of diverse stakeholders in the decision-making of ILR is a vital step in enhancing the sustainability of ILR. The aim of this paper is to use Shanghai as a case study to understand the diverse stakeholders and their participation during the decision-making of ILR in China. Interviews and questionnaires were used to collect data. Stakeholder analysis (SA) and social network analysis (SNA) were used as complementary research methodologies in this paper. First, stakeholders who participated in the decision-making of ILR were identified. Then, the characteristics of various stakeholders, including power, interests, and knowledge, were analyzed. Following this, the interactive relationships among stakeholders were explored, and their network structure was examined. Finally, policy recommendations were presented regarding stakeholder participation problems in the decision-making of ILR in China.

## 1. Introduction

Since the reform and opening up and the rapid urbanization of China, the urban population has grown rapidly [1], which has led to a significant increase in the required urban space. Urban sprawl and urban renewal are seen as two approaches to address the requirements for urban space caused by population rise. However, excessive urban sprawl can lead to a reduced agricultural land and inefficient urban land use, which is considered unsustainable [2,3]. Urban renewal can bring significant improvements in the inner city and is an effective approach to tackling urban decay and realizing multiple goals, such as improving the living conditions of citizens and the urban environment, thereby enhancing people’s well-being [4,5,6,7].

After over 40 years of rapid economic development, many large cities in China, e.g., Shanghai, Beijing, Shenzhen, and Guangzhou, have taken the lead in entering the postindustrial era, and the industrial base of these cities has shifted from manufacturing industries to service industries [8]. The majority of industrial factories located in the urban interior were moved to the edge of the city or closed, leaving a large number of vacant, inefficiently utilized industrial land plots and buildings [9]. There are two main approaches to the reuse of industrial space: adaptive reuse and industrial land redevelopment (ILR) [9,10,11]. Adaptive reuse refers to partially or completely converting the functions of the buildings on industrial site through appropriate renovation without changing the main building structure [9]. ILR refers to the overall demolition and reconstruction of industrial site that has little value and cannot be adaptively reused [10]. In China, most of the vacant, inefficiently utilized industrial sites have problematic building environment or serious safety issues such as building safety or fire safety, which can only be solved through redevelopment [12,13,14]. In Chinese practice, redevelopment is the dominant choice of decision makers, and ILR projects account for the majority of reuse of industrial space [15,16]. Take Shanghai as an example, from 2004–2018, a total of 24.20 million m^2^ of industrial buildings were demolished [17]. Therefore, the ILR project was selected as the research object of this study. ILR can bring a multitude of benefits to cities, such as increasing urban public space, improving the quality of life of citizens, improving the environment, increasing job opportunities, and promoting local economic growth [9,18,19,20]. Therefore, ILR is also an effective approach to enhance people’s well-being.

Like all complex and multidimensional public problems, ILR projects involve extensive stakeholders. Due to the lack of a comprehensive understanding of stakeholders, large-scale ILR projects often raise a series of social issues in practice, such as injustice and inequality [10,21]. Sustainable urban renewal integrates the three dimensions of facilitating economic growth, improving the environment, and enhancing social vitality [22]. With regard to the social dimension, two elements are considered in sustainability: ethical values and norms involving the extensive participation of stakeholders (for example, equity, and justice) [23]. Stakeholder participation is an effective approach to enhance sustainable urban development and has been emphasized in many studies [24,25,26]. ILR projects are public projects. Compared to the Western countries, public participation in public projects in China is relatively weak and lacks public supervision [27]. In China, the government has dominant power in public project management [28]. In practice, decisions regarding public projects are made by the government with the assistance of consulting parties, and the public is often excluded from the decision-making process [28,29]. Moreover, due to the uniqueness of institution and social culture, western lessons may not provide one-size-fits-all approach to address the problems in Chinese context [27]. Therefore, supporting stakeholder participation is always the challenging issues of public projects in China, which requires a lot of effort to investigate and resolve [27,30].

During ILR projects, the mechanism, power structures, the partnership characteristics, and the relationships among various stakeholders can have a significant influence on decision-making [6]. In effect, the stakeholder system is considered to be a collection of mutually independent individuals or organizations that are not affected by continuous interaction [31]. However, it has been stated that stakeholders in the system are generally interacting [32,33,34]. Evaluating the system contributes to promoting stakeholder participation and interactions, thereby influencing the success of the project [35]. Nevertheless, few existing studies have systematically explored the roles of diverse stakeholders and their interactions in participating in the decision-making of ILR in China. Thus, a thorough understanding of diverse stakeholders in the decision-making of ILR is essential to address social issues and thereby improve the sustainability of ILR in China.

This study aims to provide a profound understanding of stakeholders in the decision-making of ILR in China. It primarily answers three questions: Who are the stakeholders in the decision-making process of ILR? What are the characteristics of the stakeholders? Furthermore, what are the interactive relationships among the stakeholders? Shanghai, as a representative city in China and a hotspot of ILR projects, was selected as the case city for this study. The structure of this paper consists of the following. First, this paper reviews stakeholder participation in the decision-making of ILR in China. Second, the research methodology is described in this paper. Third, it analyzes the characteristics of the stakeholders and the relationships among them. Finally, the discussion and conclusions are presented.

## 2. Review of Existing Research in Decision-Making and Stakeholder Participation in ILR in China

### 2.1. Decision-Making of ILR

Over the past few decades, urban renewal has made a significant contribution in enhancing the physical environment and quality of life on a global scale, and it will continue to bring about significant changes to inner-city areas in the future [36,37]. Against the backdrop of post industrialization, the fading of urban manufacturing within intra city areas has been a problematic issue across the globe, leaving massive amounts of underutilized industrial land within cities [38,39,40]. Therefore, ILR has become a vital part of urban renewal. It is essential to meet the needs of urban economic and social development through sustainable ILR. A large number of scholars believe that ILR can bring multiple benefits, including increased job opportunities, enhanced urban image, and improved quality of life and environment [9,41]. However, it also triggers unsustainable outcomes, such as social conflicts and the destruction of industrial heritage [20,40]. To better resolve urban issues, ILR decision-making has attracted global attention in urban research. In terms of criteria and methods of decision-making, Chan et al. explore the critical factors for promoting the social sustainability of ILR in Taiwan to support better ILR decision-making [40]. Wang et al. developed a GIS-based framework that considers five land use types (including industrial land) for supporting land-use planning during urban redevelopment [42]. With regard to the decision-making behavior of stakeholders, Gao et al. apply the multistage bargaining model to analyze the logic of the decision-making behavior of key stakeholders in the redevelopment of industrial land owned by local state-owned enterprises in China and provide a reference for local authorities to propose intervention strategies [21]. Gao et al. apply multiple game models to analyze the logic behind the decision-making behavior of key stakeholders in the redevelopment of industrial land owned by central state-owned enterprises in China and provide a reference for local authorities to propose intervention strategies [43].

Although many studies and practical efforts have addressed this problem, these efforts do not always achieve positive outcomes, as it is difficult to decrease social inequality and cleavages by meeting the needs of numerous stakeholders in diverse contexts [44,45,46]. Although collaborative governance is established among government departments, residents, developers, and consultants, etc., in Western societies, such as the UK and the Netherlands, the leading cause of conflicts among stakeholders is still different discourses and the sense of inequality [47]. Compared the case in to Western countries, in China, the government’s strong power in urban land redevelopment more frequently leads to such extreme conflict situations [48]. To address these issues, understanding each stakeholder’s specific situation is the priority.

### 2.2. Stakeholder Participation in ILR Decision-Making in China

The economic development and implementation of public policy in China are greatly influenced by government intervention. The government joins market forces to realize capital accumulation via land reuse, such as ILR, influenced by both China’s socialist history and current world trends [9]. Although there have been great contributions to the development of urban society and the economy, problems also exist, such as redevelopment goals being overly focused on economic goals, the destruction of industrial heritage, and social injustice [49,50]. To facilitate stakeholder participation in urban public projects while maintaining the stability of society, the Chinese government has established some participatory consultative mechanisms, such as public hearings and consultative meetings [30]. The Chinese central government issued a reform policy in 2013 called “The Decision on Major Issues Concerning Comprehensively Deepening Reforms” [51]. This policy required a shift in the government’s role to a service-based government, devolving the power of government to nongovernment actors in public affairs. However, a universal approach to supporting stakeholder participation in the decision-making of ILR does not exist. Due to China’s specific institutions and culture, it remains a challenge to optimize stakeholder participation [12,27].

The public project’s success is built on the understanding of stakeholder interdependence [52]. Sustainable urban development must rely on the cooperation of all stakeholders and the exchange of information, targets, and resources [30]. This approach is seen to be necessary to achieve consensus and thus is widely adopted in Western countries. Drawing on this idea, a good participatory mechanism on the basis of a profound understanding of various stakeholders in China’s complex social and economic system is necessary [10,53].

Stakeholders refer to “any individual or group who can affect, or be influenced, by the organizational goals’ attainment.” [54,55,56]. According to this definition, stakeholders in ILR decision-making refer to the individuals or groups who are involved in the ILR decision-making process or who affect the decisions, and their interests are influenced by the outcome of the decisions.

Urban land ownership in China belongs to the government, and only land use rights and properties are privately owned [57,58]. Based on this, the owners of properties on industrial land are defined as the original land users in this study. In China, large-scale ILR projects can be divided into three types of redevelopment models according to the different implementation entities: state-led, land user-led, and mixed redevelopment models [59]. The state-led redevelopment model means that the government pays the original land users compensation and recovers land use rights, and there are two ways to develop the land under this model. First, the government can transfer the land use rights into the hands of state enterprises, which can develop the land individually or jointly with developers. Second, the government can sell land use rights to developers that pay land transfer fees, and the developers complete the land development. In the land user-led redevelopment model, the original land users pay the government for the land price by changing the land use or increasing the plot ratio and implement ILR in accordance with the redevelopment implementation plan formulated by the government. In practice, in most cases, large-scale ILR projects generally adopt a mixed redevelopment mode. Therefore, the mixed redevelopment model projects were selected as the research object of this study since they are more representative and generalizable.

In China, the decision-making of ILR is not a simple process. Factors such as the scope of redevelopment, redevelopment mode, redevelopment timing, urban development needs and social impact need to be considered through a complex decision-making process. Therefore, various stakeholder groups are involved in ILR decision-making process. In most instances, the government still has strong power in urban governance via its dominant control over issuing policies, allocating resources, providing services, etc. [60]. In all redevelopment models, the original land users play crucial roles in ILR, as they have the land use rights, and ILR cannot be implemented if their interests are not satisfied [21]. The public in this article refers to the low-end industrial workers, the affected surrounding residents, and the enterprise tenants. In most cases, they have little opportunity to participate in the ILR decision-making process [49]. Moreover, developers also play an essential role during the implementation of ILR projects [12]. The third parties, e.g., consultants, NGOs, and financial organizations, also make valuable contributions to decision-making; however, they are hardly discussed in existing research [61].

The characteristics of stakeholders and the relationships between them are considered an important basis for formulating policies and improving management systems [62]. With regard to stakeholder characteristics, stakeholders’ power and interest are the two basic factors in classifying them [63,64]. The former refers to the degree of influence of a stakeholder on decision-making. The latter refers to the expectations of a stakeholder as well as the degree to which these expectations are influenced by decision-making. Since ILR projects are mainly driven by a combination of government and market forces and have a significant influence on the public, the interest types of stakeholders in the decision-making of ILR in China can be classified into ”administration and politics”, “community benefits”, ”marketing performance”, or a combination of two or three the above [65,66,67]. Although the functions and goals of government departments are diverse, they all work to achieve comprehensive urban development and maintain social stability through administrative regulation, law, and policy enforcement [10]. Therefore, such interest can be defined as “administration and politics”. “Community benefits” refer to the various concerns of the affected local stakeholders, such as compensation for demolition and resettlement, living conditions, equity and justice, and employment opportunities [21,66]. “Marketing performance” means the private sector’s wish to maximize economic benefits. Furthermore, stakeholders’ knowledge about ILR decisions and the groups they belong to also greatly influence their perceptions of, and contributions to, the projects [68].

## 3. Methodology

### 3.1. Study Area

Shanghai is China’s most economically developed megacity and was one of the first cities in China to enter the postindustrial era. The decline in low-end manufacturing has led to massive amounts of low-efficiency industrial land plots within the inner city, which has become one of the crucial barriers to Shanghai’s sustainable urban development during the postindustrial era [69]. In the near future, there will be a growing number of cities in China that will enter the postindustrial era. Therefore, Shanghai’s urban development model can be considered a representative case study in China [70]. Since the Shanghai municipal government released a key policy related to ILR in 2014, ILR has come to be seen as an essential strategy for achieving sustainable urban development [1]. A total of 96 ILR projects were planned to be implemented in Shanghai from 2014 to 2016, with a total area of 628 hectares [71]. Shanghai is characterized by its massive ILR and can contribute a large number of relevant cases and resources for studying ILR. Thus, this study selects Shanghai as a representative case city of ILR in China.

Putuo District lies at the center of Shanghai. It contains extensive inefficient industrial land areas that are not suitable to meet the current requirements for socioeconomic development. Therefore, ILR is considered by the Putuo District Government as an important approach to improve the environment, enhance land use efficiency, and achieve industrial transformation and upgrading to improve the sustainable development of the area. There are 725.4 hectares of inefficient industrial land within Putuo District, which were planned to be redeveloped during 2013–2020 [72]. Compared to other districts of Shanghai, Putuo District is considered to be the hotspot of ILR. Thus, it was selected to be a representative case district for data collection.

The two methods of interviews and questionnaires were used to collect data in Putuo District, Shanghai. The data collection was focused on ILR projects within Putuo District, where many low-end industrial workers, as well as the affected surrounding residents, represent disadvantaged groups.

### 3.2. Combination of SA and SNA

ILR belongs to public project management, and stakeholder analysis (SA) and social network analysis (SNA) are two complementary methods for studying stakeholders in public project management [31,73]. Therefore, this research adopts these two research methods to achieve the research aim. SA has been increasingly applied as a research method in fields related to project management and policymaking in recent decades. This reflects managers’, policymakers’, and scholars’ recognition of the roles of key stakeholders in achieving the aim of an organization, policy direction, or project [32,74,75]. SA aims to understand the stakeholders in view of a particular organization or to determine their relevance to a system (e.g., project and policy) by analyzing their position, interest, influence, and other characteristics [32,76].

Although SA is widely adopted in many research fields, there current techniques for SA present some significant limitations [77,78,79]. SA has been criticized for its low level of practical quality and academic rigor, especially when applied in systems with a large number of stakeholders [79]. As argued by Caniato et al., in some research studies, stakeholders are often seen as isolated individuals who are not subject to continuous interaction [31]. However, from a network perspective, stakeholders are not autonomous, and their interactions are strongly related to the outcome of the project. To address the limitations of SA, more attention has been focused on SNA, which is a complementary quantitative approach that provides a valuable and feasible solution [80,81].

SNA is a research method based on the assumption that the relationships among interacting individuals are important to the system [81]. It concentrates on how stakeholders’ interactive relationships constitute a structure that can be analyzed in its own right [82]. It applies graphic theory and network models and examines the patterns that characterize the stakeholder relationships in the targeted network [83,84]. Although SNA has been widely used in several disciplines, this research method also has limitations. As Freeman argued, SNA strongly relies on mathematical models and graphical representations [85]. It can help systematically reveal the interactive relationships of stakeholders but cannot provide the many insights hidden behind the network (e.g., casual influences of the network structure).

SA addresses the characteristics of stakeholders, while SNA explores the relationship structures of stakeholders. In several studies, these two research approaches have been combined to reveal the system of stakeholders, as one method can probe the system in a way the other method cannot achieve in depth [31,78,86]. In this paper, the mixed approach of SA and SNA was adopted to enrich the integral understanding of the findings, which are explained in the discussion.

**Figure 1:**
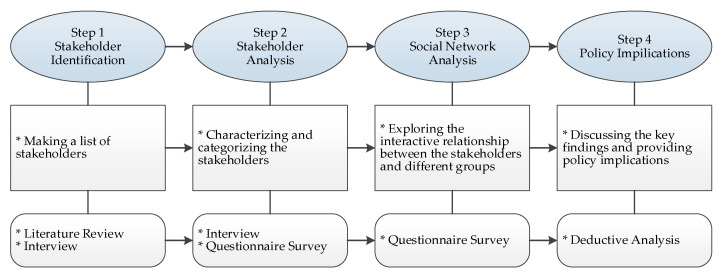
Research process.

As Figure 1 shows, this study comprises four main steps. The first step is to outline the stakeholder system and make a list of stakeholders based on a literature review and interviews. The second step is SA. Through interviews and questionnaire surveys, this step characterizes and categorizes each stakeholder based on the following characteristics: interest type, stakeholder group type, knowledge level, interest level, and power level. The third step is SNA. Through the questionnaire survey, this step probes the interactive relationships between stakeholders and different groups. Finally, the fourth step discusses the findings and provides policy implications.

For the SA, this study introduced a power and interest grid as an instrument for stakeholder assessment to map and compare the levels of power and interest across all stakeholders [62,87]. To conduct SNA, network diagrams were applied to map the stakeholders’ interactive relationships. Furthermore, the degree centrality, closeness centrality (eigenvector), and betweenness centrality of the network were calculated to analyze the network characteristics. Degree centrality is measured by the number of ties one stakeholder has with other stakeholders. It refers to the level of connectedness of the node (stakeholder) [88]. A stakeholder with a high degree centrality value can be considered more likely to obtain information and affect decisions. Nevertheless, having a high value of degree centrality does not indicate that this stakeholder has access to many other stakeholders from across the entire network. Therefore, closeness centrality (eigenvector) was utilized to describe the connections of a stakeholder across the network. It measures the degree of the connection with other stakeholders in terms of the whole network structure [89]. Moreover, to analyze a stakeholder’s control over the entire network, betweenness centrality was applied by measuring the number of shortest pathways passing through that stakeholder. A stakeholder with a high value of betweenness centrality may act as a “middleman” who can provide shorter paths of interaction between two other stakeholders [83].

### 3.3. Semi-Structured Interview

This study used semi-structured interviews to validate the stakeholder list formulated by the authors and the results of the interviews were used to deeply interpret, support and supplement the results of the SA and SNA data analysis. There were 34 stakeholders in the ILR decision-making process identified in the initial list made by the authors. The following two principles were applied in selecting the target interviewees: (1) the target interviewees were on the initial list of stakeholders, and (2) The target interviewees had experience in ILR decision-making. As it is not easy to interview each stakeholder separately (especially government departments), 27 stakeholders representing the majority of the stakeholders on the initial list in 10 stakeholder groups were ultimately contacted. These representatives include original land users, government officials, university professors, workers, citizens, and managers of real estate companies with much experience in practice or sufficient knowledge of ILR in Shanghai. The detailed background description of the interviewees is presented in Table 1.

In the interviews of this study, the interviewees were asked (1) to validate the initial stakeholder list; (2) to summarize the role of the stakeholders they represent; (3) to describe the interests of the stakeholders they represent and other stakeholders they are acquainted with, regarding administration and politics, community benefits, marketing performance, combination; (4) to summarize the collaboration and conflicts among them as well as with other stakeholders; and (5) to respond to several open-ended questions related to the obstacles or current issues in decision-making of ILR.

With the assistance of the professionals interviewed, the initial stakeholder list was adjusted, and the final stakeholder list was completed. Based on the interviews, eight more stakeholders were added to the list: two municipal government departments (Commission of Transportation and Bureau of State-owned Assets Supervision and Administration in Municipal Government), four district governmental departments (Bureau of State-owned Assets Supervision and Administration, Public Complaints and Proposals Office, Landscaping and City Appearance Administrative Bureau, and Bureau of Culture and Tourism), one organization led by district government (District Real Estate Registration Services Centre), and one consulting party (soil and water environment assessment agency). For this reason, 42 stakeholders were ultimately identified in ILR decision-making. The final stakeholder list that was verified by the interviewees is shown in Table 2.

In addition, through the interviews, it was found that although there are two redevelopment models adopted in mixed redevelopment model projects, there is currently only one decision-making process, the state-led model, in Shanghai. For this reason, there is only one stakeholder participation network involved in the decision-making in this type of project.

### 3.4. Questionnaire Survey

The questionnaires were used to collect data for SA and SNA. The questionnaire designed for this study was distributed it to the 42 stakeholders in the final stakeholder list (Table 2). The following two principles were used to select the target respondents: (1) they were among the 42 stakeholders on the final stakeholder list, and (2) they had experience of being involved in the decision-making of ILR. This study purposefully distributed the questionnaires to all stakeholders on the final stakeholder list through email and personal delivery. Eventually, 54 valid questionnaires were collected, representing all 42 stakeholders on the final stakeholder list. There are two parts of the questionnaire. The first part of the questionnaire was designed to collect data for the SA to understand the levels of knowledge, power and interest regarding ILR decision-making from the perspective of the selected stakeholders. A five-point Likert scale (0, 2.5, 5, 7.5, and 10) was used to measure the data, with 0 representing no or minimal knowledge/power/interest and 10 representing a very high level. The second part of the questionnaire was used to collect data for the SNA. In this part, the target respondents were to note all the other individuals or organizations with whom they had contact in the decision-making process of ILR.

During data analysis, the levels of knowledge, power and interest were measured by mean score X, divided into 5 groups: no or minimum (X = 0), very low (0 < X≤ 2.5), low (2.5 < X≤ 5), high (5 < X≤ 7.5), and very high (7.5 < X≤ 10). Because these factors were self-reported, the values were triangulated to examine the overall consistency through the interviews. In case of inconsistency, the authors would contact the respondents again to validate the answer. If the respondent maintained his or her views, discursive questions (for example, regarding the description of the respondent’s role and duty) were asked to verify the values. The scores of the characteristics rated by the respondents were generally consistent with the opinions of the interviewees. Moreover, this study used UCINET to analyze the interactive relationships among diverse stakeholders [90]. UCINET was applied to generate interactive networks to map the stakeholders’ connectivity and to calculate the parameters of degree centrality, closeness centrality (eigenvector) and betweenness centrality to describe the network characteristics.

## 4. Results

### 4.1. Identification of Stakeholders

**Figure 2:**
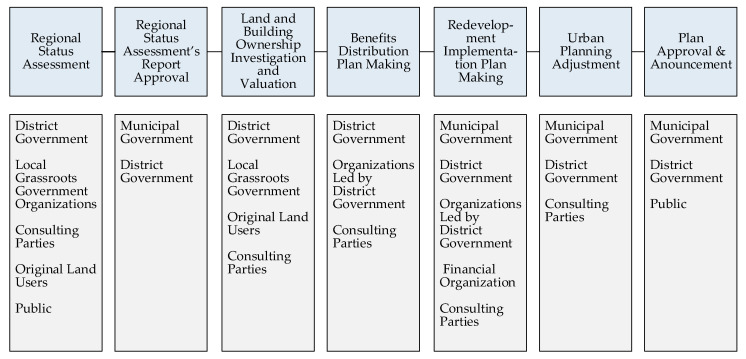
The simplified decision-making process of ILR and the stakeholder groups involved in each step of the process in Shanghai, China (by the authors).

To make a stakeholder list in the decision-making process of ILR, it is paramount to clearly understand the range of the decision-making process of ILR. The list built on the authors’ practical experience and professional knowledge is shown in Figure 2. Furthermore, the entire decision-making process can be simplified into 7 major steps.

Then, based on the interviews, 42 stakeholders from the 10 stakeholder groups in the ILR decision-making process were eventually identified. The detailed stakeholder list is shown in Table 2.

It is not the responsibility of the municipal government to initiate ILR projects in the decision-making of ILR. Conversely, the municipal government departments’ roles are primarily to formulate ILR policy, direct the work of district governments, evaluate and monitor the decision-making process, and approve final decisions. The district government, at a lower administrative level than the municipal government, takes primary responsibility for decision-making and is involved in the entire process of decision-making. Over 10 district governmental departments with land and planning, investment, industry, environment, housing, construction, financing, and other functions coordinate and cooperate in decision-making. Local grassroots government organizations are the "grassroots-level" government composed of two stakeholders, the Town Government and the Industrial Zone Management Committee. They are responsible for basic work, such as information gathering, coordination of stakeholders and policy advocacy, to support ILR decisions. Organization led by district government are state-owned organizations that resolve land development affairs. A single stakeholder represents the financial organization, the China Development Bank. In addition to financing from private enterprises and local governments, China Development Bank loans are an important funding source for ILR projects. Consultant parties are important professionals whose views are an essential basis for local governments to make decisions about ILR.

### 4.2. Analysis of Stakeholder Characteristics

**Figure 3:**
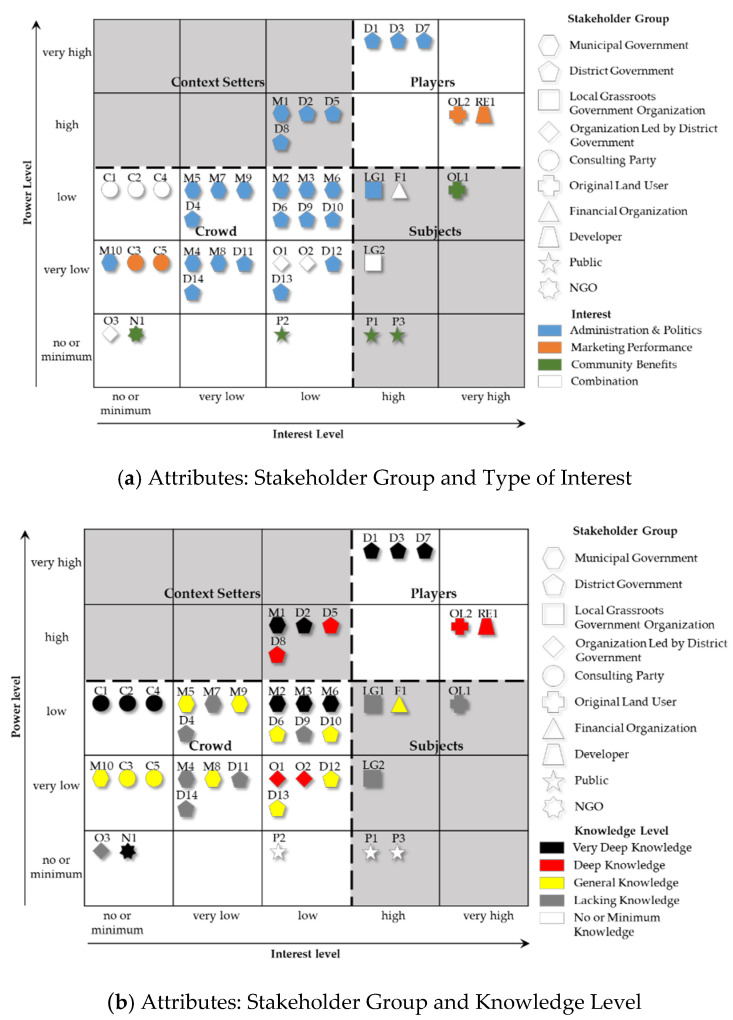
Power vs. interest grid, grouped based on (**a**) stakeholder groups and type of interest, (**b**) stakeholder groups and knowledge level.

The power and interest levels of stakeholders in the decision-making of ILR are shown in the power vs. interest grid in Figure 3, categorized by stakeholder group, knowledge level, and type of interest. The figure drawn from the questionnaire survey data is based on the mean score of each parameter for the identified 42 stakeholders. All stakeholders were categorized into four groups: players, subjects, context setters and crowd, with the mean score “5” of the power and interest levels of the stakeholders as the threshold [87]. In decision-making, the players hold strong power, and their interests are strongly influenced by the decisions. The subjects’ power in decision-making is relatively small, but their interests are strongly influenced by the decisions. In contrast, context setters hold strong power in decision-making, but their interests are little influenced by the outcome. The crowd has little influence on decisions and is not strongly influenced by the decision.

As shown in Figure 3, 25 stakeholders take “administration and politics” as their main interests, representing 3 out of the 5 players and all context setters. A total of 3 of the 5 players and 3 of the 4 context setters belong to the district government. This shows that in the decision-making process, the district government has dominant discourse power. As players, the Bureau of Planning and Natural Resources (D1), Commission of Commerce (D3), and Bureau of Housing Management (D7) are the crucial actors. As context setters, the Commission of Development and Reform (D2), Investment Promotion Office (D5) and Commission of Construction and Management (D8) are other key district government departments. These key district-level government departments synergistically make critical contributions to ILR decision-making in urban planning and land use, industry, housing, investment, and construction, separately. These departments can exert a strong influence on decisions in various aspects. Nevertheless, due to the current government accountability mechanisms, most of them are not sufficiently responsible for unintended results (Interviewees I5-D and I9-D). The Bureau of Planning and Natural Resources (M1) is the single context setter from the municipal government, and its main responsibility is for formulating ILR policies and coordinating other relevant departments of the municipal government. As the municipal government primarily plays guiding and approving roles, its influence on decisions is lower than that of the district government.

The original land users in the land user-led redevelopment model (OL2) and real estate developer (RE1) are the only two players who are not from the district government. However, compared with players from the district government, their influence on ILR decision-making is relatively low. These two stakeholders contribute to ILR through land investments. They are economically oriented stakeholders whose goals are to achieve maximized profits via ILR. The formulation of investment strategies for ILR projects relies heavily on valuable information obtained from local governments. Nevertheless, since OL2 holds land use rights and RE1 holds many resources, in most cases, they have some discourse power and can have a greater influence on decisions (Interviewees I5-D, I6-D, I7-D, I15-C, I20-OL, and I22-RE).

Subjects include six stakeholders from four different groups. Local grassroots government organizations have relatively weak discourse power in the decision-making process (Interviewees I10-LG and I11-LG). The Town Government (LG1) and the Industrial Zone Management Committee (LG2), as the grassroots government, make tremendous efforts in delivering information and coordinating conflicts among local stakeholders and other parties. They have little influence on ILR decision-making, but if the original land users and the public complain and resist decisions, they have to directly bear the impact of these consequences. The original land users in the state-led redevelopment model (OL1), low-end industrial workers (P1) and enterprise tenants (P3) are rooted in redevelopment areas. As local stakeholders, they bear the direct impact of ILR, but they have little opportunity to participate in the decision-making process (Interviewees I8-D, I16-C and I17-C). However, although they are all local stakeholders, they have different interests. Since OL1 and P3 must move their companies out of the area, their primary concern is obtaining more information and participating in the ILR decision-making process to improve economic compensation (Interviewees I11-LG, I14-C, I19-OL, and I25-P). Most of the enterprises in the ILR area are labor-intensive low-end industries, with a large number of low-end industrial workers, and most of them also rent residences in the area. When the ILR is completed, the low-end industries will be replaced by high-end industries, and the low-end industrial workers will lose their jobs and low-cost accommodation (Interviewees I11-LG, I16-C, I17-C, and I18-C). Therefore, their primary interest is to be able to obtain reasonable social security, such as economic compensation, new job opportunities or low-cost accommodation (Interviewee I24-P).

It is surprising that the consulting parties, particularly the urban planning and design agencies (C1), scholars (C2) and industrial planning agencies (C4), are stakeholders with expertise in ILR decision-making, but they are all categorized as part of the “crowd”. Their consulting services should be a critical reference for ILR decision-making. However, the research results reveal that they have little influence on the decision and are hardly influenced by the results. In many cases, the suggestions of consulting parties do not align with the desires of the local government (Interviewees I14-C, I16-C, I17-C, and I18-C). In practice, consulting parties often support the position of the local government and use their expertise to provide technical support for the government aims.

### 4.3. Network Structure

#### 4.3.1. Network Structure of the Stakeholders

**Figure 4:**
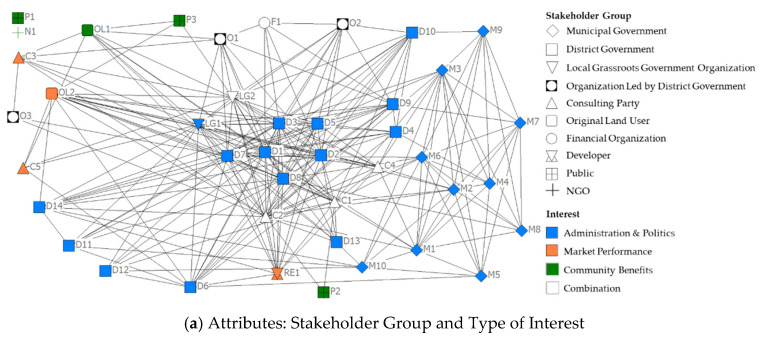

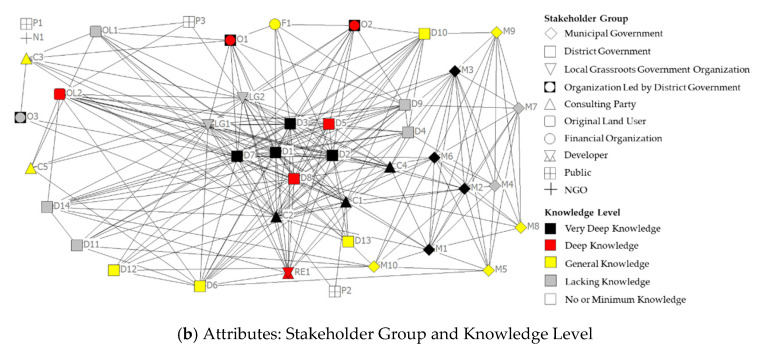
Interaction network, grouped based on (**a**) stakeholder group and type of interest, (**b**) stakeholder group and knowledge level.

The interaction network among various stakeholders in ILR decision-making is displayed in Figure 4, grouped by stakeholder group, level of knowledge and type of interest. Using the data from the questionnaire, it is possible to analyze the integration of stakeholders during ILR decisions from a network view through the connectivity of stakeholders. Figure 4 shows that no single stakeholder stands out as the dominant center of the entire network, with only a few stakeholders having fewer than five connections. Low-end industrial workers (P1) and urban renewal-related NGOs (N1) are the only two exceptions that are not connected to other stakeholders. Due to the lack of decision-making knowledge and formal participation pathways, P1 had few direct connections with others. In addition, N1 is currently only present in a few neighborhood redevelopment projects in China but is hardly involved in ILR projects (Interviewee I27-N).

The results in Figure 4 show that the stakeholders who come from district-level and municipal-level governments generally participate in a greater degree of interactions. In Shanghai, there is no single dedicated department that is fully responsible for ILR decision-making. In contrast, the main related functions are divided into a large number of departments. At the municipal and district levels of government, approximately 24 departments directly or indirectly participate in ILR decision-making. In practice, excessive separation of rights and responsibilities can lead to problems such as overlapping functions, buck-passing, difficulties in interdepartmental communication and cooperation, complex administrative approval procedures, and inefficient decision-making (Interviewees I1-M, I2-M, I5-D, I8-D, I14- C, and I16-C).

The network structure of stakeholders is also depicted by the degree, closeness and betweenness centrality of the various stakeholders and is summarized by the rankings in Table 3. The results in Table 3 reveal that none of the stakeholders can completely dominate the entire network, as the top six in the degree score ranking are relatively close in closeness centrality as well. As measured by centralities, the Bureau of Planning and Natural Resources (D1) is ranked first in both degree centrality and closeness centrality, and it is ranked third in betweenness centrality, where it can be considered to be a crucial stakeholder. Likewise, the Commission of Development and Reform (D2), Commission of Commerce (D3), Bureau of Housing Management (D7), and Commission of Construction and Management (D8) are also regarded as core stakeholders due to their high rankings. These key actors play crucial roles in both the district government and the whole network. In addition to the district government, the urban planning and design agency (C1), scholars (C2), Town Government (LG1), and Industrial Zone Management Committee (LG2) are also regarded as core roles, as they rank in the top 10 in all three centralities. Although their power in ILR decision-making is relatively weak, their connectivity demonstrates their importance within the overall network. These actors play vital roles since they are connected to many other stakeholders.

Surprisingly, the original land users in the land user-led redevelopment model (OL2) and the real estate developer (RE1) are stakeholders who can have a significant impact on decision-making, but they are not the core actors in the entire network since they are not among the top ten in the three centrality rankings. Although there are two types of redevelopment models, state-led and land user-led models, in mixed-model projects, the decision-making process of government-led model is still the only decision-making process in Shanghai (Interviewees I5-D, I6-D, I14-C, and I17-C). Based on this, the redevelopment implementation plans of all types of projects are accomplished by the district government, assisted by local grassroots government organizations and consultants. OL2 are not allowed to make redevelopment implementation plans by themselves and can only express their willingness and interests to the government in the decision-making process, so they are not connected with many stakeholders. However, the lack of decision-making procedures for different project types can lead to problems, such as increased government workloads, inefficient decision-making, and difficulty in mobilizing OL2 to implement ILR (Interviewees I5-D, I6-D, I14-C, and I15-C).

Formally, to prevent social conflicts, developers are not allowed to be involved in the decisions. However, it is common for local governments to communicate informally with developers during the decision-making process to ensure that at least one developer will bid for the industrial land. In fact, it is common for developers to reach agreements with the government before the final decisions are completed (Interviewees I5-D, I6-D, I15-C, I22-RE, and I23-RE). Based on this, RE1 only has connections with stakeholders such as the core departments of district government, key consulting parties, and local grassroots government organizations in decision-making, so there is not very high connectivity in the network.

#### 4.3.2. Network Structure of Different Groups

As shown in Figure 4, the network of stakeholder groups is well communicated and connected overall, but there are still many important stakeholder groups that are not connected to each other. For instance, important stakeholder groups, such as noncore sectors of the district government, developers, original land users, organization led by district government, and the public, are hardly directly connected to each other. This can result in the above-mentioned important stakeholder groups not communicating effectively in decision-making, which can lead to problems such as increased conflict and difficulty in reaching consensus (Interviewees I5-D, I9-D, I10-LG, I13-O, and I15-C, I20-OL).

To obtain a better understanding of the relationships in the network of decision-making from an overall perspective, group centralities are used in describing the network characteristics of different groups. The three types of centrality measures are summarized by the type of stakeholder group, type of interest and level of knowledge, as shown in Table 4.

Among the stakeholder group types, local grassroots government organizations are well connected in the network. However, the district government, the stakeholder group with the highest power, scored lower than local grassroots government organizations in all three centralities, mainly since the functions of the district government are divided into more than a dozen departments. As the district government is the main body responsible for decision making, the excessive division of functions leads not only to difficulties in coordination and cooperation within district government departments but also to difficulties in communication and cooperation between government departments and other key stakeholders, thus reducing the efficiency of decision making (Interviewees I5-D, I7-D, I10-LG, I14-C, I17-C, I20-OL, and I23-RE). In addition, the consulting party can be seen as a “broker” of information, as it ranks second in betweenness centrality. In comparison, stakeholder groups, such as municipal government, original land users, and developers, are relatively peripheral within the network. However, with the exception of NGOs, which do not participate, the public scored the lowest in the three centralities, indicating that this group is completely marginalized in decision-making and has hardly any influence on decision-making.

Regarding interest types, there is no doubt that the interest of administration and politics has a dominant position within the entire network, as it scores highest in degree centrality. Moreover, the results show that the combination of interests is also crucial throughout the network. However, all actors within this stakeholder group are either government-affiliated organizations or third parties under the direction of the government. In comparison, market performance is relatively peripheral within the network. Community interests are completely marginalized in the network. Although ILR is aimed at benefiting the public (Interviewees I1-M, I2-M, I3-M, I4-M, I5-D, I6-D, I7-D, I8-D, and I9-D), stakeholders who represent the community benefits do not play a core role in the network.

As for the level of knowledge, it is obvious that stakeholders with very deep knowledge about decision making occupy a central position within the whole network. This stakeholder group scores first in both degree centrality and betweenness centrality. Group with deep knowledge and group with lacking knowledge also have a high value in the three centralities, indicating that they also belong to critical parts of the network. With the exception of the group with lacking knowledge, the knowledge level of stakeholders is positively correlated with their level of involvement. In addition, the group with no or minimum knowledge consists of all the public stakeholders, who has the lowest level of involvement in decision-making. The public’s extremely low level of knowledge about decision making, which leads to insufficient participation ability, is currently one of the crucial obstacles to effective public participation (Interviewees I1-M, I4-M, I5-D, I8-D, I14-C, and I15-C).

## 5. Discussion

### 5.1. Stakeholder Participation from the Perspective of SA and SNA

This study shows that SA and SNA are complementary. Combining these two approaches can provide an in-depth understanding of stakeholder participation in ILR decision-making. SA is the most advanced tool of non-technical valuation procedures [31]. It exposes the knowledge, power, and interest structure of the stakeholders during the decision-making process by taking into consideration the identified stakeholders. In this study, the SA indicates that stakeholders with the interest of “administration and politics”, particularly the district government, dominate the discourse in decision-making. In addition, although the original land users in the land user-led redevelopment model (OL2) and real estate developers (RE1), who represent the interest of “market performance”, are not decision makers, they can still have a significant influence on decisions through the resources they hold. Most local stakeholders represent “community interests”, which are strongly influenced by the decisions. However, with the exception of OL2, the other local stakeholders have little influence on the decisions. Moreover, as the stakeholders having “very deep knowledge”, it is surprising that consulting parties have little influence on the decisions and are hardly influenced by the consequences. This result is in contrast with the results of much previous research on public projects and policies, which indicate that the services of consulting parties are considered a critical basis for decision-making [91,92,93].

In this study, SNA also strengthens the results obtained through SA and contributes complementary results via a quantitative and graphical view. Although local grassroots government organizations have little power in decisions, they have many connections with the other stakeholders. This implies that they play a significant role in decision making by cooperating and coordinating. Although OL2 and RE1, as stakeholders that can have a significant impact on decision-making, they are not the core actors in the entire network. Municipal government sectors are in the highest administrative level of decision-making, but they are relatively peripheral to the entire network, and they primarily play a role in guiding the work of district governments and approving final decisions. Furthermore, although the government reported that the purpose of ILR is to benefit the public, the results show that the public not only has extremely little power in decision-making but is also completely marginalized in the network and has no influence on decision-making. The heterogeneous and complex network of interaction is more completely revealed for better scrutiny, and its meaning can be better understood via SNA. The network’s heterogeneity and complexity lead to interaction, information exchange, and collaboration. As Sandström and Carlsson argue, the high degree of interaction between stakeholders can promote communication, reduce conflicts and facilitate joint action, particularly when there are numerous connections among different types of stakeholders [94]. Nevertheless, this can also result in the disaffection of many stakeholders. Therefore, it may decrease the probability of action by crucial stakeholders, as these stakeholders must satisfy many others [95].

The combined application of SA and SNA brings additional benefits, especially providing a more in-depth understanding of the decision-making of ILR in China. The results of this study provide not only an integral picture of the stakeholder system but also a detailed assessment of the issues of stakeholder participation. Thus, it is clear that combining the application of these two methods has better implications for approaches to address related issues and enhance stakeholder systems.

### 5.2. The Complexity of Local Government Departments

As the findings of this study reveal, the complexity of interactions among government stakeholders is evident. In China, although the government has dominant power in ILR decision-making, many government departments at diverse administrative levels are involved in ILR projects. However, the functions and responsibilities of government departments are not clearly defined when they cooperate in decision-making. Based on this, although some core government departments can significantly influence decisions on urban planning and land use, industry, housing, investment, etc., separately, none of them are completely responsible for the project’s success, nor does any department have the power to control the overall picture. ILR decision-making is a complex process. The complexity of the government sector causes not only many problems within the government sectors but also difficulties and inefficiencies in cooperation between the government and other stakeholders. Unsurprisingly, Huxham, Vangen, Huxham, and Eden believe that "tangles of ties" can also result in "partnership fatigue", reducing transparency, accountability and restricting contact with the outside world [96].

If we want to compare with this study of mainland China with studies in two Asian regions, Hong Kong and Singapore, for example, the majority of urban renewal affairs are undertaken by a dedicated department (the Urban Renewal Authority in Hong Kong and the Urban Redevelopment Authority in Singapore) [97]. The establishment of a dedicated department can significantly improve the efficiency of decisions and solve the problems of overlapping functions and mutual shirking of responsibility etc., which are commonly encountered in large bureaucratic and hierarchical governments. Therefore, the concentration of functions and powers of ILR into fewer departments can become a model for China to follow.

### 5.3. Irrational Decision-Making Procedures

In China, developers’ participation in land expropriation and house demolition has caused frequent social conflicts and has been criticized by some studies [98,99]. In 2011, the central government issued a new law, “the Regulation on the Expropriation and Compensation of Houses on State-owned Land”. It prohibits developers from participating in land acquisition and housing demolition, and developers can bid for land use rights only after the government has completed the aforementioned work [99]. Based on this, land acquisition and housing demolition in the ILR are undertaken and compensated by the government alone, which greatly increases the workload and financial pressure on local governments. Since the land user-led redevelopment model does not require land acquisition and has multiple benefits, such as reducing social conflicts, reducing the government redevelopment workload and alleviating financial pressure, some cities in China have begun to explore and encourage using this model to promote ILR [100]. The consideration of multiple types of redevelopment models in parallel are of great significance to the smooth promotion of ILR. However, there is currently only one decision-making process, the state-led model, in which all types of projects’ decisions are organized and completed by the government, and this significantly increases the government’s workload in decision-making, and thus reduces the efficiency of decision-making for all types of projects. In addition, the original land users in the land user-led redevelopment model also face problems such as excessively long decision-making time, leading to increased capital cost risks and difficulties in meeting their needs in the implementation plan formulated by the government, which greatly reduces their incentive to implement ILR.

Comparing mainland China and Taipei, it is found that in urban redevelopment projects in Taipei, two different decision-making procedures are designed according to the different types of implementation entities: the state-led model and the landowner-led model [101]. The design of these different types of decision-making processes makes it possible to make decisions more efficiently and better meet the needs of different implementation entities, making this an effective model worthy of reference in mainland China.

### 5.4. Obstacles to Communication between Stakeholder Groups

De Nooy believes that high-level interaction and communication between stakeholder groups can reduce misunderstandings and prevent conflicts, which is beneficial to reaching consensus among stakeholder groups [102]. However, the results show that in the ILR decision-making process in China, many important stakeholder groups have almost no direct connection with each other, which poses an obstacle to communication between them. To transfer information and coordinate interests among the above stakeholders in the decision-making process, the only way is to rely on the certain stakeholder groups, such as core departments of the district government, consulting parties and local grassroots government organizations. This can prevent the above important stakeholder groups from effectively communicating in the decision-making process, which can lead to problems such as increased conflict and difficulties in reaching consensus among stakeholders. As Warner states, a multi-stakeholder platform can effectively increase the connections and interactions between stakeholders, enabling more effective communication between stakeholders [103]. Based on this, it is necessary to build a multi-stakeholder platform within the ILR decision-making process in China to improve communication among stakeholder groups and thus facilitate cooperation.

### 5.5. Barriers to Protecting Public Interest

#### 5.5.1. Failure to take Social Responsibility

Xian and Chen argue that people’s quality of life and social costs should be taken into account when formulating revitalization policies for cities that need to renovate their industrial spaces [104]. Nevertheless, the results show that the public, as the most fundamental interest group in ILR, is excluded from decision making, which often leads to the neglect of public interests. Especially for the low-end industrial workers, they will lose their jobs and low-cost accommodation when an industrial site was selected for redevelopment. According to the interviews in this study, in all redevelopment models, only the original land users in the state-led redevelopment model and the enterprise tenants can obtain compensation in land acquisition and housing demolition, while the dismissed low-end industrial workers are left without corresponding compensation. This is because the implementing entities (governments or the original land users in the land user-led redevelopment model) do not take up social responsibilities and disregard the needs of these low-end industrial workers since they want to minimize the compensation in land acquisition and housing demolition. As a result, the interests of these vulnerable groups without property rights and discourse are sacrificed; however, this issue rarely receives attention in existing studies. To achieve sustainable ILR, the interests of different stakeholders, especially vulnerable groups, should be taken into consideration.

#### 5.5.2. The Challenges to Effective Public Participation

In addition to the failure for implementing entities to take up social responsibilities observed in this study, the lack of public participation is another barrier to protecting the public interest in China and has been criticized by many studies [29,60,105]. In Western countries, extensive public participation has been recognized as a key success factor for public projects [106,107]. Thus, in view of the situation in China, scholars believe that technical and institutional measures, such as adding more approaches for public participation and empowering the public, can address this problem [53,108,109]. However, this study believes that only introducing the above measures will not necessarily achieve better results, given the finding that effective public participation also faces two other challenges: the insufficient participation ability of the public and the lack of trust between the public and decision-makers. The result shows that the public are stakeholders with “no or minimum knowledge”, which means that they lack expertise related to decision-making and therefore lack the ability to participate in decision-making. In addition, through the interviews in this study, the authors found that there is a lack of trust between the public and decision-makers in ILR. Due to negative past experiences with the government’s lack of responsiveness to the public interests, the public has little trust in decision-makers. Therefore, members of the public believe that even if their participation were increased, they would not have much influence on decision-making. Nevertheless, from the viewpoint of professionals, such as consultants and the government, the current awareness and participation ability of the public in decision-making in China have not yet reached the level required for deep participation in decision-making. Professionals argue that the public awareness is still relatively weak, as individuals focus only on what is directly related to their economic interests and pay insufficient attention to broader interests, such as regional development, history and culture. Furthermore, professionals believe that the public lacks knowledge and organized participation related to decision-making, and an overemphasis on in-depth public participation will greatly reduce the efficiency of decision-making. Therefore, without enhancing the participation ability of the public and improving trust between the public and decision-makers, it will be difficult to achieve effective public participation.

Many studies believe that NGOs in public projects can not only enhance the public’s awareness and participation ability but also play a bridging role in increasing trust between the public and decision-makers, which can enhance the effectiveness of public participation [110,111,112]. The involvement of NGOs has been observed in a few neighborhood redevelopment projects in China and has contributed to effective public participation to some extent [113]. However, there is no involvement of NGOs in ILR projects yet, and therefore it is necessary to cultivate NGOs related to ILR.

### 5.6. The Need for Specialized Laws and Regulations and Accountability

In the interviews in this study, many professionals frequently referred to specialized laws and regulations, as well as accountability. In the absence of specialized laws and regulations regarding the decision-making of ILR, the powers, responsibilities, functions, rights, and interests of the various stakeholders cannot be clearly defined. Lack of clarity in the powers, responsibilities, and functions of different stakeholders not only causes many problems among government departments but also leads to difficulties in stakeholder cooperation as well as weak discourse power of the public and third parties. In addition, the lack of clarity of the rights and interests of different stakeholders can allow implementing entities in ILR to avoid being proactive in taking their social responsibilities, thus ignoring the interests of vulnerable groups. Lack of reasonable accountability makes it possible for stakeholders with strong power to influence decision-making without regard for the unintended outcomes by their actions. Cheung and Leung argue that a reasonable government accountability mechanism can improve the satisfaction of stakeholders, particularly vulnerable groups [114]. In ILR, accountability mechanisms can reinforce the government’s responsibilities and enhance its willingness to cooperate with vulnerable groups.

## 6. Conclusions

ILR is an effective approach to enhance people’s well-being. However, due to the lack of a comprehensive understanding of stakeholders, large-scale ILR projects often raise a series of social issues in practice. Therefore, this study explores the characteristics of stakeholders and their interactive relationships during ILR decision-making in Shanghai, China. The results demonstrate that the joint use of SA and SNA in urban research in the context of China can provide a better evaluation and understanding of stakeholders within the overall system. Putuo District of Shanghai is selected as the study area for this study, and 42 stakeholders are identified. The results of this study reveal a high degree of complexity in the characteristics of stakeholders and the network of interactions among them in the decision-making process of ILR. The government plays a dominant role in the decision-making regarding ILR in China. However, the excessive number of government departments at diverse administrative levels participating in the decision-making process poses significant obstacles to stakeholder cooperation. The presence of multiple types of redevelopment models in parallel can reduce the government’s redevelopment workload and financial pressure. Nevertheless, in the current institution, the lack of multiple-type decision-making procedures has become one cause of project implementation failure for various redevelopment models. In addition, many important stakeholder groups have almost no direct connection with each other in the network, causing difficulties in communication between them. The failure of the implementing entities to take up social responsibilities and the lack of public participation are considered two major barriers to protecting the public interest. Regarding public participation, only introducing technical and institutional measures, such as increasing participation approaches and empowering the public, may not be fully successful, as effective public participation also faces two other current challenges: the insufficient participation ability of the public and the lack of trust between the public and decision-makers.

With regard to stakeholder participation, the concentration of government functions and powers into fewer key departments is a top priority, as such an effort can make the administration of ILR more efficient. In addition, different decision-making processes can be designed for different types of redevelopment models to improve the efficiency of decision-making for all types of projects. Moreover, a multi-stakeholder platform can be established to facilitate effective communication between stakeholder groups. Furthermore, it is necessary to cultivate NGOs to improve the participation ability of the public and increase trust between the public and decision-makers to achieve effective public participation. There is also a need to formulate specialized laws and regulations regarding ILR to clearly define the powers, functions, rights and interests of various stakeholders. Finally, a reasonable government accountability mechanism should be established to improve the government’s responsibilities and enhance its willingness to cooperate with vulnerable groups.

This study selects Shanghai as the case study area since it is the representative city of ILR in China. Therefore, this study not only provide guidance for ILR practices in Shanghai, but also for many other cities in China. In terms of the cities outside China, although the results of this study cannot be directly adopted due to the uniqueness of institution, culture, and society, the complementary methods of SA and SNA can be applied for studying the ILR issue in local context. Due to the limitation of time and resources, this study can only conduct SA and SNA in view of the whole decision-making. Future research will probe the issues by considering the dynamics of different decision-making stages. Furthermore, on the basis of this study, a framework for decision-making regarding ILR can be developed to improve participatory ILR.

## Figures and Tables

**Table 1:** Detailed background description of the interviewees.

Stakeholder Group	ID	Role or Position	Description of Departmental Function and Qualification
Municipal Government	I1-M	Government Officeholder	Work at Bureau of Planning and Natural Resources; expert in land resources management, more than 15 years of work experience with ILR projects
	I2-M	Government Officeholder	Work at Commission of Economy and Informatization; urban industrial development specialist, more than 10 years of work experience with ILR projects
	I3-M	Government Officeholder	Work at Commission of Development and Reform; expert in urban economics, more than 10 years of work experience with ILR projects
	I4-M	Government Officeholder	Work at Commission of Housing and Urban-Rural Construction Management; more than 15 years of work experience with ILR projects
District Government	I5-D	Government Officeholder	Work at Bureau of Planning and Natural Resources; expert in land resources management, more than 15 years of work experience in the urban land use management of ILR projects
	I6-D	Government Officeholder	Work at Commission of Commerce; urban industrial development specialist, more than 15 years of work experience with ILR projects
	I7-D	Government Officeholder	Work at Commission of Development and Reform; expert in urban economics, more than 10 years of work experience with ILR projects
	I8-D	Government Officeholder	Work at Bureau of Housing Management; more than 15 years of work experience with urban land expropriation
	I9-D	Government Officeholder	Work at Commission of Construction and Management; more than 10 years of work experience with ILR projects
Local Grassroots Government Organization	I10-LG	Deputy Mayor	Work at Town Government; grassroots work expert for ILR projects
	I11-LG	Deputy Director	Work at Industrial Zone Management Committee; grassroots work expert in ILR projects
Organization Led by District Government	I12-O	Deputy Director	Work at District Land Reserve Center; more than 10 years of work experience in urban land management of ILR projects
	I13-O	Manager	Work at District Government-affiliated Platform Company; more than 15 years of work experience in urban land development of ILR projects
Consulting Party	I14-C	Urban Planner	Work at an urban planning and design agency; more than 10 years of practical experience in ILR projects
	I15-C	Professor	Work at the university; more than 15 years of experience in research and practice of ILR projects.
	I16-C	Professor	Work at the university; more than 10 years of experience in research and practice of ILR projects.
	I17-C	Researcher	Work at an urban research institute; researcher of ILR and conservation of industrial heritage
	I18-C	Director	Work at an industrial planning agency; more than 10 years of practical experience in ILR projects
Original Land User	I19-OL	Entrepreneur	Original land user in state-led redevelopment model
	I20-OL	Entrepreneur	Original land user in land user-led redevelopment model
Financial Organization	I21-F	Officer	Work at Shanghai Branch of China Development Bank; more than 10 years of work experience in investment in ILR projects
Developer	I22-RE	Manager	Work at a real estate development company; more than 15 years of work experience in urban land development of ILR projects
	I23-RE	Manager	Work at a real estate development company; more than 10 years of work experience in urban land development of ILR projects
Public	I24-P	Worker	A migrant worker in an industrial site that will be redeveloped
	I25-P	Manager	A manager of an enterprise tenant in an industrial site that will be redeveloped
	I26-P	Citizen	A resident around an industrial site that will be redeveloped
NGO	I27-N	Officer	Working at an urban renewal-related nongovernmental organization; more than 10 years of work of experience in urban renewal

**Table 2:** Stakeholders and their groups in industrial land redevelopment (ILR) decision-making in Shanghai, China.

Group	Stakeholder	
Municipal Government	M1. Bureau of Planning and Natural Resources	M2. Commission of Development and Reform
	M3. Commission of Economy and Informatization	M4. Commission of Science and Technology
	M5. Bureau of Ecology and Environment	M6. Commission of Housing and Urban-Rural Construction Management
	M7. Bureau of Finance	M8. Commission of Transportation ^1^
	M9. Bureau of State-owned Assets Supervision and Administration ^1^	M10. Other Specific Sectors
District Government	D1. Bureau of Planning and Natural Resources	D2. Commission of Development and Reform
	D3. Commission of Commerce	D4. Commission of Science and Technology
	D5. Investment Promotion Office	D6. Bureau of Ecology and Environment
	D7. Bureau of Housing Management	D8. Commission of Construction and Management
	D9. Bureau of Finance	D10. Bureau of State-owned Assets Supervision and Administration ^1^
	D11.Public Complaints and Proposals Office ^1^	D12. Landscaping and City Appearance Administrative Bureau ^1^
	D13. Bureau of Culture and Tourism ^1^	D14. Other Specific Sectors
Local Grassroots Government Organization	LG1. Town Government	LG2.Industrial Zone Management Committee
Organization Led by District Government	O1. District Land Reserve Center	O2. District Government-affiliated Platform Company
	O3. Real Estate Registration Services Centre ^1^	
Consulting Party	C1. Urban Planning and Design Agency	C2. Scholar
	C3. Real Estate Assessment Company	C4. Industrial Planning Agency
	C5. Soil and Water Environment Assessment Agency ^1^	
Original Land User	OL1. Original Land User in State-led Redevelopment Model	OL2. Original Land User in Land User-led Redevelopment Model
Financial Organization	F1. China Development Bank	
Developer	RE1. Real Estate Developer	
Public	P1. Low-end Industrial Worker	P2. Affected Surrounding Resident
	P3. Enterprise Tenant	
NGO	N1.Urban Renewal-related NGO	

^1^ Indicates that these stakeholders were added to the list based on the interviews.

**Table 3:** The centrality measures of various stakeholders.

Code ^1^	Degree Centrality		Closeness Centrality		Betweenness Centrality	
	Degree	Rank	Eigenvector	Rank	Betweenness	Rank
D1	25.000	**1**	0.276	**1**	43.206	**3**
D7	25.000	**1**	0.268	**2**	54.417	**2**
C2	24.000	**3**	0.251	**6**	75.422	**1**
LG1	23.000	**4**	0.256	**5**	32.152	**6**
D3	22.000	**5**	0.257	**3**	25.747	**9**
D2	22.000	**5**	0.257	**3**	26.957	**8**
D8	21.000	**7**	0.243	**7**	37.197	**5**
C1	20.000	**8**	0.217	**10**	41.818	**4**
LG2	19.000	**9**	0.220	**9**	31.251	**7**
D5	18.000	**10**	0.227	**8**	10.337	19
D6	15.000	11	0.173	14	25.089	**10**
D9	15.000	11	0.181	13	19.809	14
OL2	15.000	11	0.191	11	20.184	13
C4	14.000	14	0.172	15	9.657	22
M3	14.000	14	0.104	24	23.228	11
M2	13.000	16	0.091	28	18.603	16
M6	13.000	16	0.096	25	22.348	12
M1	13.000	16	0.092	27	19.270	15
RE1	13.000	16	0.186	12	1.305	35
D10	13.000	16	0.169	16	15.012	17
D14	12.000	21	0.160	17	8.507	23
O2	11.000	22	0.152	18	1.991	33
O1	10.000	23	0.123	20	4.789	26
D4	10.000	23	0.126	19	9.732	21
OL1	10.000	23	0.111	22	9.804	20
M10	9.000	26	0.056	32	13.229	18
M7	9.000	26	0.046	33	5.077	25
D11	8.000	28	0.105	23	3.211	29
D13	8.000	28	0.115	21	3.651	28
M9	8.000	28	0.043	36	3.122	31
M4	8.000	28	0.040	37	3.654	27
M5	8.000	28	0.044	35	5.853	24
D12	7.000	33	0.093	26	3.135	30
C3	7.000	33	0.079	29	2.289	32
M8	7.000	33	0.045	34	1.962	34
F1	6.000	36	0.078	30	0.274	37
C5	5.000	37	0.059	31	0.583	36
O3	3.000	38	0.037	38	0.000	39
P3	3.000	38	0.033	39	0.125	38
P2	2.000	40	0.029	40	0.000	39
P1	0.000	41	0.000	41	0.000	39
N1	0.000	41	0.000	42	0.000	39

The bold numbers represent the top 10 stakeholders in each type of centrality ranking. ^1^ Note: D1 = Bureau of Planning and Natural Resources, D2 = Commission of Development and Reform, D3 = Commission of Commerce, D7 = Bureau of housing management, D8 = Commission of Construction and Management, LG1 = Town Government, LG2 = Industrial Zone Management Committee, C1 = Urban Planning and Design Agency, C2 = Scholar.

**Table 4:** Group centrality measures.

Category	Group	Degree	Closeness (Eigenvector)	Betweenness
Type of Stakeholder Group	Municipal Government	10.200	0.066	11.635
	District Government	15.786	0.189	20.429
	Local Administrative Organization	21.000	0.238	31.702
	Organization Led by District Government	8.000	0.104	2.260
	Consulting Party	14.000	0.156	26.098
	Original Land User	12.500	0.151	14.994
	Financial Institution	6.000	0.078	0.274
	Developer	13.000	0.186	1.305
	Public	1.667	0.021	0.042
	NGO	0.000	0.000	0.000
Type of Interest	Administration & Politics	13.840	0.143	17.380
	Marketing Performance	11.667	0.129	6.271
	Community Benefits	3.000	0.035	1.986
	Combination	13.375	0.156	20.650
Knowledge Level	Very Deep	17.083	0.173	30.056
	Deep	14.667	0.187	12.634
	General	8.455	0.087	6.811
	Lacking	11.700	0.128	12.320
	No or Minimum	1.667	0.021	0.042
